# Tele-Simulated Instruction and Learner Perceptions of Fiberoptic Intubation and Nasopharyngoscopy: A Pilot Study

**DOI:** 10.5811/westjem.2022.11.58053

**Published:** 2022-12-21

**Authors:** Andrew D. Bloom, Rachel E. Aliotta, Alexander Mihas, Dawn Taylor Peterson, Derek A. Robinett, Marjorie Lee White

**Affiliations:** * University of Alabama at Birmingham, Department of Emergency Medicine, Birmingham, Alabama; † University of Alabama at Birmingham, Department of Orthopaedic Surgery, Birmingham, Alabama; ‡ University of Alabama at Birmingham, Department of Medical Education, Birmingham, Alabama; § University of Alabama at Birmingham, Department of Pediatrics, Birmingham, Alabama

## BACKGROUND

The coronavirus disease 2019 (COVID-19) pandemic presented many challenges to medical training and education. As the number of cases grew worldwide, medical schools and institutions were forced to cease in-person activities to limit disease spread. The immediate result was the cancellation of conferences and didactics, temporarily halting medical education. As a response, distance-based, virtual learning rapidly popularized. Adapting in-person lecture learning styles to a remote digital setting has been proven acceptable as a new educational platform.^1^ However, with this transition to digital learning, many distancing guidelines paused or limited in-person, hands-on simulations.

Today, simulation-based training continues to be a well-established and integral part of medical education.^2^ Simulation allows trainees to practice technical and non-technical skills in a safe environment without risk to patients. It has frequently been demonstrated that simulation effectively promotes skill acquisition and translates to improved patient care outcomes.^3^–^6^ In emergency medicine (EM), simulation-based training is essential to meet Accreditation Council for Graduate Medical Education (ACGME) requirements.**7** Simulation affords trainees increased exposure, practice, and competency in rare and infrequently encountered procedures**.8** Importantly, progressing medical trainee’s knowledge and skills during periods of distancing regulations requires the development of new and effective strategies to deliver simulation and high-quality medical and procedural training.^8^,^9^

In the emergency department, airway management is a critical skill to master. Increasing patient complexity, physiology, and anatomy have necessitated the need for adjunct and alternative strategies to manage more difficult airways. Fiberoptic nasopharyngoscopy can serve as a life-saving airway skill.^10^–^16^ Allowing visualization of laryngeal anatomy provides valuable diagnostic information in angioedema, airway obstruction, and many other head and neck airway emergencies. While intubations are frequently encountered in practice, fiberoptic nasopharyngoscopy is far less often used but extremely useful, making it ideal for simulation-based training.

## OBJECTIVES

The objective of our study was to evaluate learners’ self-reported comfort and competency after learning advanced airway procedures including fiberoptic intubation and nasopharyngoscopy in a telesimulation vs standard in-person simulation setting.

## CURRICULAR DESIGN

We conducted a prospective, randomized pilot study approved by the institutional review board in spring 2021 in a university simulation center. The type of procedural instruction was chosen based on a questionnaire sent to all resident classes identifying fiberoptic intubation and nasopharyngoscopy as the most requested and least performed procedure. The curriculum was developed using Sawyer’s learn, see, practice, prove, do, maintain (LSPPDM) framework for procedural skill training in medicine.^17^

Study participants were EM residents from all three trainee years in the emergency department at a large level I trauma center-university hospital system. All participants had at least completed a four-week rotation in anesthesia, and successfully performed multiple orotracheal intubations. The EM residents voluntarily agreed to participate in the study and provided informed consent. One week before the study began, all participants received a formal, slideshow-based lecture during scheduled didactics on fiberoptic nasopharyngeal intubation and were provided instructional, web-based videos for viewing. No prior hands-on instruction was given.

After formal instruction the simulations were held during weekly protected didactics time. The study was conducted over three consecutive weeks with each participant assigning themselves to a 20-minute timeslot online. This was contingent upon the work shift-education availability of the individual resident. The facilitator was a board-certified emergency physician with fellowship training in medical simulation.

Participants arrived in succession at their individual assigned timeslots and completed a pre-intervention survey to assess perceptions, comfort, and self-perceived ability to perform the advanced airway procedure. The survey also included previous experience, preparedness, and attitudes toward the procedure in the ED. The survey used a Likert scale of 1–10 (1 - not comfortable, to 10 - extremely comfortable) for all qualitative questions. Afterward, they were randomized to either the standard simulation (SIM) group or virtual tele-simulation (Tele-SIM) group with a random number generator without repeats. Even-numbered participants were assigned the SIM group and odd Tele-SIM, respectively.

After randomization, the facilitator arrived in person with the SIM participants, while the Tele-SIM participants would be introduced to the same facilitator at a remote location via live video broadcast. Participants were then given an identical short, pre-brief introduction to the single-use flexible bronchoscope, Ambu aScope Broncho Slim (Ambu Inc, Columbia, MD), and intubation supplies and adjuncts. The participants were taught proper handling of the scope technique for proper nasopharyngoscopy, as well as strategies for troubleshooting. Participants were then asked to perform nasopharyngoscopy with the device to properly visualize the vocal cords. They then performed nasotracheal intubation using the scope. The facilitator was present to assist, answer questions, and troubleshoot any complications either in person for the SIM group or virtually via live video broadcast. After successful nasotracheal intubation the participants were given time to perform the procedure again, with facilitator support as needed during the 15-minute session. The remaining five minutes were spent preparing for the next participant.

**Figure 1:**
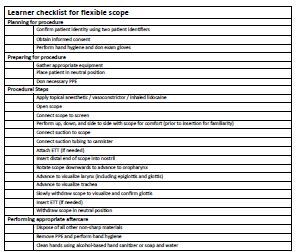
Learner checklist for flexible scope. *PPE*, personal protective equipment; *ETT*, endotracheal tube.

The participants were observed and rated by the facilitator using a checklist (Figure 1). The checklist outlined proper handling of the broncho*scope*, adequate manipulation of the device, and troubleshooting measures. The checklist was developed and reviewed by emergency physicians and intensivists at our institution who regularly perform the procedure. The facilitator was not blinded to the study hypothesis. Upon completion of the procedural component, all participants were individually asked to complete a post-simulation survey. Those in the Tele-SIM cohort were then given the opportunity to receive in-person instruction and facilitation if they wished with any remaining time.

**Figure 2:**
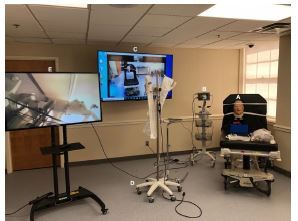
Room for emergency medicine resident learner. Manikin head for nasopharyngoscopy and intubation (A); fiberoptic flexible bronchoscope (B) connected to monitor (E); Zoom projection of facilitator in nearby location (C); additional intubation scope for teaching and debriefing (D); and projected fiberoptic camera for ease of facilitator visualization (E).

To provide an adequate and similar experience for the Tele-SIM group a series of audiovisual equipment was set up (Figure 2). Each group was provided identical equipment to perform the procedure including but not limited to the scope, airway manikin, endotracheal tubes, and elastic gum bougie. In the participants’ room two monitors were set up. The first monitor projected the facilitator in a nearby room broadcasted via Zoom (Zoom Video Communication, San Jose, CA]. The second monitor was connected to the scope to allow the facilitator visualization of the participant’s technique (Figure 2). This allowed timely feedback while the procedure was being completed.

In a nearby room the facilitator had available the same supplies as the participant. The facilitator was in front of a large screen projecting themselves and their image to the participant in the first room. This allowed for any real-time instruction on handling the scope or troubleshooting. Full live audio was available to both the facilitator and the participant for the entirety of the simulation and teaching experience.

The design for the Tele-SIM group was developed with assistance from our simulation center. In the weeks leading up to the study, EM faculty and simulation staff determined best practices for the video session based on trial and error. Prior to the study we participated in multiple walk-through sessions to identify issues and improve overall flow.

## IMPACT/EFFECTIVENESS

Overall, 28 EM residents of various postgraduate year (PGY) training levels from PGY 1–3 participated in the training. The number of times using a flexible scope before training and the number who had any formal training before the exercise were similar between the two groups (Table). Residents in both Tele-SIM and SIM learning groups reported a self-perceived improvement in their scope intubation skills after their simulation and training session (Table). Both groups felt increased comfort with the scope as well as their ability to visualize the anatomy of the laryngopharynx/vocal cords (LP/VC) with the scope (Table). There was no significant difference in improvement between the two groups. Both groups felt more comfortable to teach this procedure to their peers and felt increased utility in their clinical practice in the ED following completion (Table).

The learner’s ability and confidence in procedural competency increased significantly in both groups. Most importantly, all learners felt more comfortable operating the scope and performing this advanced procedure afterward with Tele-SIM group pre: 2.4 +/− 1.9 improving to post: 7.9 +/−1.0, and the SIM group pre: 2.6+/− 1.2 improving to post: 7.5+/− 1.5 as well (Table 1). There was no significant difference comparing the two groups across pre- and post-survey domains.

Both groups felt the goals of the simulation were clear and they had adequate supervision during the simulation. Specifically, the Tele-SIM group felt they had adequate support and that the teaching modality complemented their learning style. No Tele-SIM learners crossed over and requested additional training session time in person when offered. All learners regardless of simulation technique felt they were given the tools they needed to succeed and were satisfied with their simulation experience (Table 1).

The COVID-19 pandemic has highlighted the need for continued efforts in procedural simulation delivery and medical education. With limits on in-person instruction, telesimulation can serve as an additional tool for teaching both from a procedural and non-procedural aspect. From the perception of our learners, our study suggests real-time instruction can be similarly meaningful and supportive to increase learner comfort whether presented virtually or in person, even for a complex airway procedure. Here we observed no differences or limitations between reported learner experiences.

To help bridge the gap of needed simulation-based training and in-person limitations, telesimulation has been increasingly used. Telesimulation was defined by McCoy et al as a process by which telecommunication and simulation resources are used to provide education, training, or assessment to learners at an offsite location.^18^ Telesimulation in procedural task training has been studied in areas such as surgery, ultrasound, and intraosseous needle placement, focusing mostly on training in remote and resource-limited regions.^19^–^23^ There has also been increasing use of telesimulation in non-procedural, case-based situations with promising results.^24^,^25^

While initially developed to provide learning to resource-limited areas, telesimulation in the era of COVID-19 has also been studied.^26^,^27^ Most studies have focused on case-based, non-procedural simulation. Despite its ongoing use, there exists a paucity of literature comparing telesimulation to conventional simulation in a prospective manner. To our knowledge there are no studies that have evaluated advanced procedural airway training delivered virtually vs in person. If successfully implemented in airway training and other procedures, telesimulation could maintain learners’ health and safety without sacrificing important training experiences.

In development of a novel procedural telesimulation, we realized the importance of a precise set-up. Multiple monitors were required to project images back to the facilitator, particularly to capture detailed imaging. High speed internet was essential for in-time audio and visual feedback without delay. Finally, a larger space with adequate soundproofing helps prevent echoing and any interference for the learner and facilitator.

Here our data supports learners’ perceived satisfaction with telesimulation as an alternative or adjunct to standard simulation, a modality that can extend far beyond the limitations of the COVID-19 era. Both groups self-reported improvement in performance, knowledge, and comfort after participating in the simulation with no significant difference between the two groups.

The primary limitation of this pilot study is that our findings, while encouraging, consist of opinions and self-perceived competency. While learners reported similar procedural comfort, it is not clear whether their skills truly improved. Further study is needed with objective outcomes measuring procedural skills to show that telesimulation is indeed a comparable learning experience. This study also represents a single encounter with the learner. Additional research is needed to evaluate learner retention and skill decay over time but can only be achieved after objective skills are measured. Resource-limited areas without access to such technology may create difficulties in delivery. Having only one instructor present limited objective evaluation of participants. The results represent a single institutional experience with a small sample size and are limited to a sole procedure being simulated and taught. Future studies may look to validate telesimulation in delivery style as well as across different procedure simulation categories.

As the educational landscape continues to adapt to social limitations and embrace distance-learning structures, consideration should be taken to implement telesimulation for teaching critical hands-on procedures and skills to future emergency physicians.

## Figures and Tables

**Table:** Group characteristics and pre- and post-simulated reported experiences.

Pre-simulation group characteristics	Tele-SIM (n=14)	SIM (n=14)	P-Value
PGY Level			0.129
PGY-1	7 (50.0%)	2 (14.3%)	
PGY-2	4 (28.6%)	7 (50.0%)	
PGY-3	3 (21.4%)	5 (35.7%)	
Prior to workshop: how many times used scope			0.454
0	8 (57.1%)	6 (42.9%)	
1 to 3	5 (35.7%)	7 (50.0%)	
4 to 6	0 (0.0%)	1 (7.1%)	
>6	1 (7.1%)	0 (0.0%)	

Mean reported pre- and post-simulation experience comfort scores	Tele-SIM (n=14)	SIM (n=14)	P-Value

Perceived comfort level operating scope			
Pre-workshop	3.2 ± 2.2	3.2 ± 1.6	0.949
Post-workshop	7.9 ± 1.1	8.0 ± 1.5	
Perceived comfort visualizing airway anatomy with scope			
Pre-workshop	3.6 ± 2.1	3.4 ± 1.7	0.911
Post-workshop	8.6 ± 1.0	8.4 ± 1.4	
Perceived comfort level performing scope assisted intubation			
Pre-workshop	2.4 ± 1.9	2.6 ± 1.2	0.824
Post-workshop	7.9 ± 1.0	7.5 ± 1.5	
Perceived comfort teaching peers procedure			
Pre-workshop	1.9 ± 1.4	1.6 ± 0.8	0.796
Post-workshop	7.4 ± 1.5	7.4 ± 1.3	
Feel scope is useful skill in practice			
Pre-workshop	8.0 ± 2.7	7.6 ± 3.3	0.829
Post-workshop	9.9 ± 0.4	10.0 ± 0.0	

Post workshop learner experience survey	Tele-SIM	SIM	P-Value

The goals of simulation were clearly outlined prior to participation	9.3 ± 1.1	9.7 ± 0.7	0.352
Felt had enough supervision during simulation	9.6 ± 0.7	10.0 ± 0.0	0.352
Felt comfortable asking question or for help during simulation	9.9 ± 0.5	10.0 ± 0.0	0.769
Felt was given adequate feedback during simulation	9.7 ± 0.6	10.0 ± 0.0	0.352
Simulation complemented learning style	9.7 ± 0.6	10.0 ± 0.0	0.352
Perceived knowledge of indications, and procedural technique improved	9.7 ± 0.5	9.9 ± 0.4	0.541
This workshop would be useful for future ED clinicians to participate	9.6 ± 1.1	10.0 ± 0.0	0.541
I am satisfied with overall simulation experience	9.6 ± 0.9	10.0 ± 0.0	0.352

*PGY*, postgraduate year.
